# Binaural audio frontend processing for cochlear implants inspired by the medial olivocochlear reflex

**DOI:** 10.3389/fnins.2025.1678288

**Published:** 2025-11-28

**Authors:** Enrique A. Lopez-Poveda, Almudena Eustaquio-Martín, Milagros J. Fumero, Reinhold Schatzer, Joshua S. Stohl, Christian Wirtz, Peter Nopp

**Affiliations:** 1 Instituto de Neurociencias de Castilla y León (INCYL), Universidad de Salamanca, Salamanca, Spain; 2 Instituto de Investigación Biomédica de Salamanca (IBSAL), Universidad de Salamanca, Salamanca, Spain; 3 Departamento de Cirugía, Facultad de Medicina, Universidad de Salamanca, Salamanca, Spain; 4 MED-EL GmbH, Innsbruck, Austria; 5 North American Research Laboratory, MED-EL Corporation, Durham, NC, United States

**Keywords:** cochlear implants, audio coding and processing, speech in noise recognition, sound localization, medial olivocochlear reflex, hearing loss

## Abstract

**Objectives:**

The “MOC strategy” is a binaural audio processing method for cochlear implants (CI) inspired by the medial olivocochlear (MOC) reflex. Although the strategy can improve hearing for CI users, it requires a bilateral channel pairing for the contralateral control of the backend compression and, therefore, the same number of frequency channels and electrodes on both sides. This may limit its clinical applicability. As a step to overcome this difficulty, here, we present and test a version of the MOC strategy designed to operate at the front end (FE) rather than the back end of processing, referred to as the MOC_FE_.

**Design:**

The MOC_FE_ strategy was implemented and tested in combination with a pair of functionally independent bilateral MED-EL FS4 audio processors. The hearing of bilateral CI users was compared for the MOC_FE_ as well as for two reference strategies: the standard FS4 strategy and a backend MOC strategy. The MOC_FE_ and MOC strategies were implemented with fast contralateral inhibition. Measures included (1) speech reception thresholds for sentences in fluctuating and stationary noise, in unilateral and bilateral listening modes, and for three different speech levels; and (2) sound source localization in quiet and in noise. Five bilateral users of MED-EL CIs participated in the evaluations.

**Results:**

(1) For speech intelligibility in fluctuating noise, the MOC_FE_ was as beneficial (re FS4) as the backend MOC strategy, except for one specific spatial configuration (S_−60_N_60_), where the MOC_FE_ produced no benefits while the MOC strategy did. (2) Neither the MOC_FE_ nor the MOC strategies improved intelligibility in a stationary noise, possibly because they involved fast rather than slow contralateral inhibition. (3) The binaural MOC_FE_ and MOC strategies tended to improve sound source localization slightly relative to the FS4 strategy.

**Conclusion:**

The MOC_FE_ can become a successful alternative to the MOC strategy, as it may be more easily implemented in clinical devices and applied to a broader range of clinical map configurations.

## 1 Introduction

Cochlear implants (CIs) are an effective treatment for many people with severe-to-profound hearing loss (Zeng, 2022). Not only do current CIs restore a sense of hearing, but they can also provide effective speech intelligibility in quiet backgrounds for most users (Boisvert et al., 2020). Unfortunately, CI users still find it harder than normal to understand speech in noisy settings even when they use two devices, one per ear (Loizou et al., 2009). Here, we present and test a binaural audio frontend processing method intended to improve speech-in-noise intelligibility for users of bilateral CIs.

Many everyday listening situations involve understanding the speech from a target speaker among a myriad competing speakers or noise sources. People with normal hearing accomplish this task more easily when listening with two ears than when listening with either ear alone (Loizou et al., 2009). However, the benefit of listening with two ears rather than one is less or zero for CI users. There are various possible reasons why bilateral CIs fall short of providing normal speech intelligibility in noisy settings or in “cocktail party” listening scenarios. These include (but are not limited to) the use of two independently functioning CIs with different number of frequency channels, misaligned electrodes, and/or different acoustic-to-electric maps, all of which can distort or degrade binaural acoustic cues (e.g., Litovsky et al., 2012; Jones et al., 2014; Kan, 2018). In this regard, the use of binaurally coupled CI audio processors might improve the benefits from bilateral cochlear implantation.

**Figure 1:**
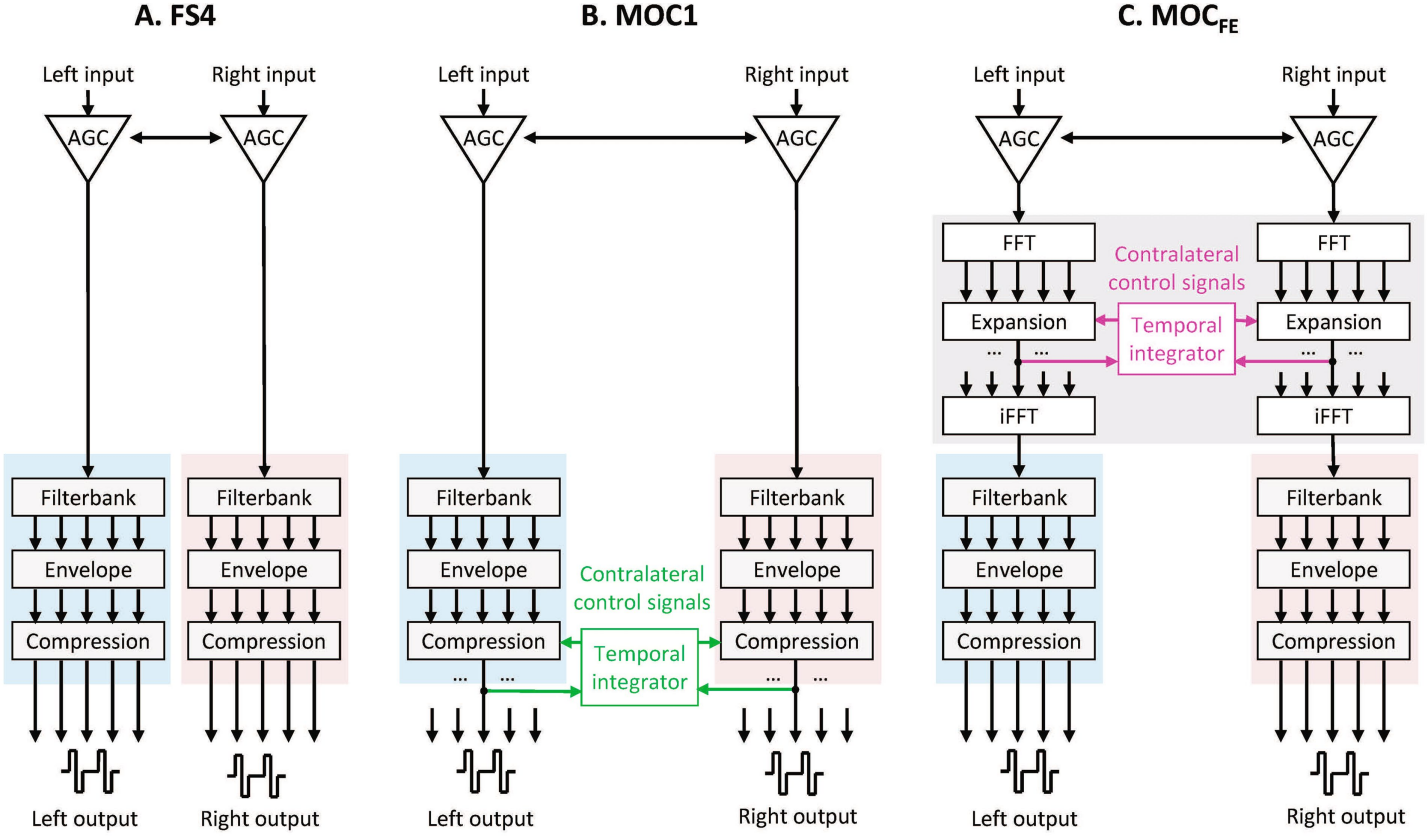
Processing strategies. **(A)** FS4: binaurally linked AGC followed by two functionally independent MED-EL’s temporal fine-structure processors, one per ear. **(B)** MOC1 strategy: linked AGC followed by an FS4 strategy in each ear with dynamic, contralateral control of backend compression. **(C)** MOC_FE_ strategy: linked AGC followed by contralateral control of expansion followed by two functionally independent FS4 processors with fixed backend compression.

We have previously shown that the intelligibility of speech in competition with other sounds can be improved by using a binaural CI sound-coding strategy inspired by the contralateral medial olivocochlear reflex (MOCR) (Lopez-Poveda et al., 2016a, 2017). The processing strategy was named “the MOC strategy.” Just like in the healthy ear cochlear mechanical gain and compression is modulated by contralateral sounds (reviewed by Lopez-Poveda, 2018), in the MOC strategy the compressive acoustic-to-electric map of each CI varies dynamically (in time) depending on the output from the contralateral CI (see Figure 1B and the text below). Compared to using two independently functioning CI audio processors, the MOC strategy can enhance amplitude modulations and the head shadow (Lopez-Poveda et al., 2016a) and thereby improve the speech information in the acoustically better ear (Lopez-Poveda and Eustaquio-Martín, 2018). As a result, the MOC strategy can improve the intelligibility of speech in noise (Lopez-Poveda et al., 2016a, 2017, 2020; Fumero et al., 2021) with a mild positive impact on sound localization (Lopez-Poveda et al., 2019). Those results suggest that the MOC strategy holds promise for improving outcomes for CI users.

The MOC strategy, however, may have limits in its clinical applicability. Because the strategy involves dynamic (time varying) contralateral control of the acoustic-to-electric maps and these maps are typically applied at the back end of processing (Wilson et al., 1991; Wouters et al., 2015), the implementation of the MOC strategy in hardware would require to couple pairs of identical channels/filter banks on the two sides and, therefore, the same number of active electrodes in both CIs, which is rare in clinical BiCI users. As with most other assistive listening devices, CIs include means to preprocess the audio input to enhance desired features (e.g., to improve the signal-to-noise ratio) before it is subject to standard CI processing (cf. Figure 1 of Wouters et al., 2015). To facilitate the implementation of the MOC strategy in clinical CIs, it would be preferred that the MOC strategy, or a version of it, be implemented as preprocessing, i.e., in the audio domain and at the front end (FE) of processing (Figure 1). This would provide a channel specific bilateral coupling independent of the clinical mapping on the left and right side. Therefore, the MOC concept could be employed by all bilateral CI users with their own clinical maps.

As a step to facilitate a potential implementation of the MOC strategy in clinical devices, the aim of the present study is to present and experimentally test a frequency-domain, FE version of the MOC strategy, termed “MOC_FE_ strategy.” This strategy was implemented in combination with MED-EL’s temporal fine-structure processing (FS4) and evaluated experimentally for five users of bilateral CIs. The evaluation included (1) speech-in-noise intelligibility for various speech levels, for steady and fluctuating noises, and for multiple spatial configurations of the speech and noise sources; and (2) localization of sound sources in quiet and in competition with a noise source. For reference, performance was also measured with a standard FS4 strategy (i.e., two independently functioning FS4 audio processors) and with a backend MOC strategy with fast contralateral control of compression. For consistency with the nomenclature used in previous studies (Lopez-Poveda and Eustaquio-Martín, 2018; Lopez-Poveda et al., 2020), the latter will be referred to as the MOC1 strategy. We hypothesized that performance with the MOC_FE_ would be better than with the reference FS4 strategy, and comparable to that with the MOC1 strategy.

## 2 Materials and methods

### 2.1 Processing strategies

Stimuli were processed through three processing strategies: FS4, MOC1, and MOC_FE_. All three strategies were implemented with binaurally linked automatic gain control (AGC), which was placed at the far front of processing (Figure 1). The linked AGC applied level-dependent identical broadband gain at the two ears and equal to the minimum gain across the ears (Wiggins and Seeber, 2013).

#### 2.1.1 The FS4 strategy

The FS4 strategy (Figure 1A) intends to preserve the temporal fine structure (TFS) cues in speech (e.g., Zierhofer, 2001; Riss et al., 2008; Schatzer et al., 2010). This strategy involved two functionally independent FS4 sound processors, one per ear. Each processor included a high-frequency pre-emphasis filter; a bank of time-domain bandpass filters with a modified logarithmic distribution between 70 and 8,500 Hz; envelope extraction via Hilbert transform; a logarithmic compression function (Equation 1 in Lopez-Poveda et al., 2016a); and sampling of compressed envelopes with biphasic electrical pulses using the FS4 approach, i.e., using channel-specific sampling sequences in the four most apical channels and time-interleaved fixed-rate stimulation sequences in the remaining channels (for more details about the FS4 strategy, see Riss et al., 2014). The number of filters in the filter banks were identical to the minimum number of active electrodes between the left and right implants (Table 1), and equal for the left-ear and right-ear processors. The reason for this was to have identical channel/filter banks configurations for the FS4 and MOC_FE_ strategies as for the MOC1 strategy, where same number of channels/filter banks is a necessity. This allows a fair comparison between strategies, so any effect of processing strategy on patient performance will be due to the use of dynamic compression (MOC1) or expansion (MOC_FE_) rather than to other factors like the number of channels.

**Table 1:** Participant information.

ID	Sex	Age (years)	Etiology	Time of implant use (months)		Processor/implant/electrode array in the clinical devices		Processing strategy in the clinical devices		Electrodes active/used for testing		Pulse rate (pps)		Better ear	*c* value in the clinical devices		THR (%MCL)	
				L	R	L	R	L	R	L	R	L	R		L	R	L	R
S1	M	53	Mg	232	247	OPUS2 C40+ Standard	RONDO SONATAti100 FLEXsoft	HDCIS	FS4-p	1–9 1–9	1–7,9–11 1–7,9–10	1,500	1,322	L	900	500	10	10
S2	F	40	Mg	203	75	RONDO PULSAR ci100 Standard	RONDO CONCERTO FLEX28	FS4-p	FS4-p	1–11 1–11	1–12 1–11	1,200	1,600	L	1,000	500	10	10
S3	M	51	Mg	194	209	RONDO PULSAR ci100 Standard	RONDO PULSAR ci100 Standard	FS4-p	FS4-p	1–11 1–11	1–12 1–11	1,210	1,277	L	500	500	10	10
S4	F	79	Un	203	173	SONET PULSAR ci100 Standard	SONET PULSAR ci100 Standard	FS4-p	FS4-p	1–10 1–10	1–2,4–11 1–2,4–11	2,100	1,268	L	500	500	10	10
S5	F	44	Un	80	227	RONDO CONCERTO FLEX28	RONDO CONCERTO FLEX28	FSP	FS4-p	1–12 1–12	1–12 1–12	1,230	1,293	L	500	500	10	10

*c*: backend compression parameter value in the participants clinical CIs. F, female; L, left; M, male; Mg, meningitis; pps, pulses per second; R, right; THR, threshold; Un, unknown.

The backend compression function (or acoustic-to-electric map) was as follows (Boyd, 2006):


$$y=\frac{\log(1+\mathrm{xc})}{\log(1+c)},$$ (1)

Where *x* and *y* are the input and output amplitudes to/from the compression function, respectively, and *c* is a parameter that determines the amount of compression. In the FS4 strategy, the value of *c* was set equal to 1,000 and was fixed. This value differed slightly from the value of 500 used by some of the participants in their clinical devices (Table 1). As discussed by Lopez-Poveda et al. (2020, p. 1507), this difference is unlikely to have a major impact on performance or on the benefit of MOC processing.

Note that the FS4 strategy was regarded as the reference strategy because it was the closest to the strategy employed by the participants in their clinical devices, except for the use of a linked AGC (note that the number of active channels and frequency allocation on the two sides departed from the clinical values in some patients) (Table 1).

#### 2.1.2 The MOC1 strategy

The MOC1 strategy is a binaural, signal processing method to mimic the effects of the contralateral MOCR with CIs at the back end of processing (Figure 1B) (Lopez-Poveda, 2014; Lopez-Poveda et al., 2016a). The MOC1 strategy was like the FS4 strategy in all aspects except that the value of the compression parameter (*c* in Equation 1) in every frequency channel varied dynamically depending upon the time-weighted output level from the corresponding frequency channel in the contralateral processor. The relationship between the instantaneous value of *c* and the instantaneous contralateral output level (*E*) was such that the greater the output level, the smaller the value of *c* (on-frequency inhibition) (Figure 2 in Lopez-Poveda et al., 2016a). The contralateral output level was calculated over an exponentially decaying time window with two time constants (*τ_a_* and *τ_b_*). The contralateral control of compression was identical as described in former publications (Lopez-Poveda, 2014; Lopez-Poveda et al., 2016a, 2016b, 2017), where further details can be found. Here the two time constants were set equal to 2 ms, i.e., with fast contralateral control of compression. This form of control is expected to improve intelligibility more in fluctuating than in the steady noise (Lopez-Poveda et al., 2020).

#### 2.1.3 The MOC_FE_ strategy

The MOC_FE_ strategy is a binaural, audio processing method designed to mimic the effects of the contralateral MOCR with CIs at the front end rather than the back end of processing. In other words, the MOC_FE_ is intended as a frontend analogue of the MOC strategy (compare Figure 1C with Figure 1B). Conceptually, the MOC_FE_ consists of having dynamic contralateral controlled expansion at the front end of processing such that when combined with fixed compressive acoustic-to-electric maps at the back end, operates likes dynamic contralaterally controlled backend acoustic-to-electric maps.

The MOC_FE_ was implemented in the frequency domain as follows. The time-domain output signal from each AGC in the pair was first subject to a standard frame-based, time-overlapping fast Fourier transform (FFT) to obtain the signal frequency spectrum for each frame.

This amplitude spectrum was attenuated (“expanded”) by the inverse of the backend compression function (maplaw), i.e., by the inverse of Equation 1:


$$Y=\frac{{\left(1+C\right)}^{X}-1}{C}$$ (2)

Where *X* and *Y* (note the use of capital letters) are the single-sided amplitude spectrum of the input and output signals to the attenuator, respectively, and *C* is a parameter that determines the amount of attenuation applied. Notice that the attenuation was applied to the amplitude spectrum and that the phase (angle) was preserved.

Like in the MOC strategy, where the compression parameter *c* varies dynamically depending on the output contralateral level, in the MOC_FE_, the attenuation parameter *C* also varied dynamically, on a frame-by-frame basis, depending upon the frame-weighted (time-weighted) output level for the corresponding frequency in the contralateral processor, *E*_FE_. Note that the range of contralateral output levels, *E*_FE_, used to calculate the *C* parameter in the MOC_FE_ strategy is different from the range of output levels, *E*, used to calculate the *c* parameter in the MOC strategy. In the MOC strategy the contralateral output levels (*E*) are the levels at the output of the backend compression while in the MOC_FE_ strategy the contralateral output levels (*E*_FE_) are calculated at the output of the expansion (compare Figure 1C with Figure 1B).

**Figure 2:**
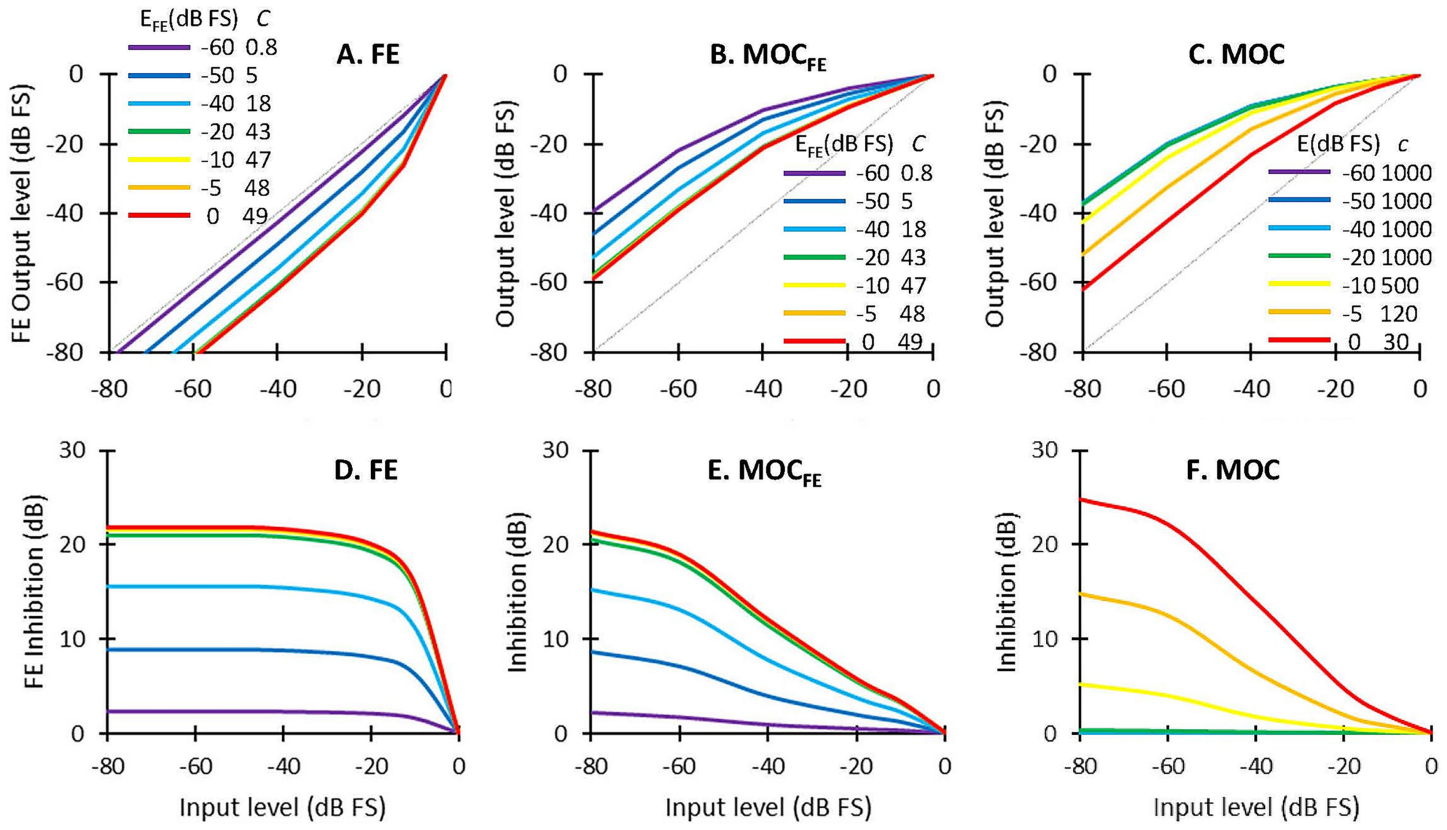
Example input/output (I/O) curves for the FE expansion, the MOC_FE_, and the MOC1 strategy. **(A)** I/O functions for the FE expansion (Equation 2) for various contralateral output levels (*E*_FE_) and corresponding values of parameter *C*. **(B)** I/O curves for MOC_FE_ strategy for different contralateral output levels (*E*_FE_) and corresponding values of *C*. **(C)** I/O curves for the MOC1 strategy for different contralateral output levels (*E*) and corresponding values of *c*. **(D)** FE inhibition (in dB) as a function of input level for the values of *E_FE_* shown in **(A)**. **(E,F)** Inhibition (in dB) as a function of input level corresponding to panels **(B,C)**. Inhibition is calculated relative to the control condition without a contralateral signal (i.e., with *E* = *E*_FE_ = −∞ dB, and *c* = 1,000 and *C* ~ 0.0001). Note that in panels **(A,B,D,E)** lines with similar *C* parameter are overlapped (green to red). In panels **(C,F)** the four lines with equal *c* parameter also overlap (purple to green).

The relationship between the value of *C* for any given frame and the contralateral output level from the frontend processor (*E*_FE_) was such that the greater the output level, the greater the value of *C* (on-frequency inhibition) (Figure 2A). Like in the MOC1 strategy, the contralateral output level was calculated over an exponentially decaying time window with two-time constants (*τ_a_* = *τ_b_* = 2 ms).

Figure 2A illustrates input/output (I/O) curves for the FE expansion function (Equation 2) for different contralateral output levels, *E*_FE_, and corresponding values of *C* parameter. Note that lines with similar *C* values (between 43 and 49) are overlapped (green to red). Figure 2B illustrates I/O curves for the FE expansion function combined with fixed backend compression (with *c* = 1,000). For reference, Figure 2C illustrates the shapes of the compression function (Equation 1) for different values of the contralateral output level, *E*, and corresponding values of *c* parameter. Note that all lines for *c* = 1,000 are overlapped and illustrate the compression function of the FS4 strategy. Figures 2D–F shows corresponding inhibition curves where inhibition is defined as the output level in dB relative to the output in the absence of a contralateral stimulus (i.e., with *E* and *E*_FE_ equal to minus infinity dB). Note that the compression and the inhibition in the MOC_FE_ strategy (i.e., with contralaterally controlled expansion at the front end combined with fixed backend compression) are roughly like the contralaterally controlled backend compression in the MOC1 strategy; that is, for both the MOC1 and MOC_FE_ strategies, the greater the contralateral output level (*E*_FE_ in the MOC_FE_ or *E* in the MOC1 strategy), the greater the inhibition. Note, however, that the I/O and inhibition curves are different for the MOC_FE_ and MOC1 strategies because in these examples the values of *E*_FE_ and *E* were chosen to be equal rather than as they would correspond for a given input audio.

In the MOC_FE_ strategy, the contralateral control could be applied on a per frequency basis. In that case, the implementation of the MOC_FE_ strategy would involve the binaural exchange of as many values of the contralateral output level *E*_FE_ as there are frequencies in the FFT. In principle, the exchange would occur at most once per frame. However, the parameter *C*, was calculated grouping the FFT frequency bins in frequency bands according to a logarithmic distribution like the backend MOC, where logarithmically distributed frequency bands are coupled. While in the backend MOC the number of bands must be equal to the minimum number of active electrodes in each side, in the frontend MOC the number of bands can be set independently from the number of active electrodes.

### 2.2 Participants

Five bilateral users of MED-EL CIs participated in the study (Table 1). All participants reported that they performed very well with their implants. Participants were volunteers and not paid for their time, but their travel and accommodation expenses were reimbursed, and they were paid a daily allowance. They all signed an informed consent to participate in the study. All participants were native speakers of Castilian Spanish. All of them (except S5) had been tested in the laboratory with the MOC1 strategy, without the linked AGC, at least one year before the present study. The study was approved by the Ethics Review Board of the University of Salamanca.

### 2.3 Fitting and loudness level balancing

Before any testing, the electrical current levels at the maximum comfortable loudness (MCL) were measured using the method of adjustment. Minimum stimulation levels (i.e., thresholds) were set to 10% of MCL values (Boyd, 2006). Processor volumes were set independently for each ear and for each processing strategy to ensure that a sentence from a source at 0° azimuth and 0° elevation was perceived as comfortable, in the center of the head, and equally loud across strategies. Final volumes are shown in Table 2. Once set, thresholds, MCL levels, and volumes remained constant for each participant across test conditions. We note that volumes were higher for the MOC1 and MOC_FE_ strategies than for the FS4 strategy. This is (presumably) to compensate for the reduction of audibility caused by the mutual inhibition between the pairs MOC1 and MOC_FE_ processors (Figures 2E,F).

**Table 2:** Speech stimuli and processor volumes used to test each participant.

		Volume (%)					
Participant	Speech material	FS4		MOC1		MOC_FE_	
		L	R	L	R	L	R
S1	Matrix sentences	80	80	85	85	100	95
S2	Matrix sentences	80	80	85	85	95	95
S3	Matrix sentences	75	70	80	75	95	90
S4	HINT sentences	70	75	85	90	100	105
S5	HINT sentences	70	70	85	85	95	95

Matrix, Spanish matrix sentences; HINT, hearing-in-noise test sentences; L, left; R, right. Note that S4 and S5 were tested with HINT rather than matrix sentences because they could barely recognize matrix sentences in quiet.

### 2.4 Equipment and virtual acoustics

The MATLAB software environment (R2019b, The Mathworks Inc.) was used to perform all signal processing and implement all test procedures, including the presentation of electric stimuli. Monophonic waveforms were first linearly scaled to the desired level. Here, levels are expressed in decibels relative to a maximum amplitude of unity and denoted as dB full scale (FS). The scaled, monophonic waveforms were convolved through appropriate head-related impulse responses (HRIRs) to obtain the unprocessed waveforms at the left and right ears for the desired (simulated) free-field target-masker spatial configuration. The resulting signals were processed through the corresponding strategy, and the resulting electrical stimulation patterns delivered using the MAX Interface Box (MED-EL cochlear implant programing box) and each patient’s implanted receiver/stimulator(s).

Spatial configurations were achieved by convolving monophonic recordings with HRIRs obtained with the front microphone of a behind-the-ear MED-EL SONNET sound processor placed on an artificial head and torso (G.R.A.S. 45BB-2). Responses were recorded in a low-reverberation chamber (RT60 = 0.06 s, direct-to-reverberant ratio = 13 dB). The levels of the stimuli were determined using the broadband RMS amplitude and were set before HRIR convolution to preserve head-related cues.

Because all stimuli were directly delivered to the participant’s implant, sound insulation was not necessary. For this reason, during the measurements, participants were seated in a regular room, and the experimenter was typically sitting in front of the participant controlling the experimental software and scoring the participant’s responses if appropriate.

### 2.5 Speech reception thresholds

The method was virtually like in our former related publications (e.g., Fumero et al., 2021).

#### 2.5.1 Procedure

Speech intelligibility in noise was assessed by measuring the signal-to-noise ratio (SNR) at which participants recognized 50% of the sentences that were presented. This will be referred to as the speech reception thresholds (SRT). SRTs were measured using fixed-level speech and varying the noise level adaptively using a one-down, one-up procedure (Levitt, 1971). To measure each SRT, we presented 30 sentences to the participants, who were asked to repeat what they heard. The experimenter scored each sentence as correct or incorrect before presenting the next sentence. A “correct” score implied that the participant recognized all words in the sentence, while an “incorrect” score implied that the participant did not recognize one or more words. Participants did not receive feedback on their responses. The first 10 sentences were always the same (taken from a practice list) but were presented in random order. This allowed participants to become familiar with the processing strategy tested during the corresponding SRT measurement. The initial SNR was 20 dB, and it changed in 3-dB steps for the first 14 sentences and 2-dB steps for the final 17 sentences. The SRT was calculated as the mean of the final 17 SNRs (the 31st SNR was calculated and used in the mean but not actually presented). Three SRTs were measured for each test condition, and the mean was regarded as the final SRT. During the experiment, the presentation of each sentence was controlled by the experimenter.

#### 2.5.2 Test conditions

Speech reception thresholds in noise were measured in two listening conditions: bilaterally (i.e., with the two CIs) and unilaterally with the self-reported better ear. (The choice of presenting the stimulus to the self-reported better ear was arbitrary.) In all cases, the speech and noise sources were at eye level (0° elevation). Locations were chosen so that the speech source was always in front or toward the self-reported better ear of each participant. The better ear reported by all the participants was the left ear (Table 1). The noise was always in front or toward the self-reported worse ear. In bilateral listening for speech at −38 dB FS, SRTs were measured for four spatial configurations of the target and masker sources (S_0_N_90_, S_0_N_0_, S_−15_N_15_, and S_−60_N_60_). In bilateral listening for speech level at −28 and −48 dB FS, SRTs were measured for three target-masker spatial configurations (S_0_N_0_, S_−15_N_15_, and S_−60_N_60_). In unilateral listening with speech at −38 dB FS, SRTs were measured for two target-masker spatial configurations (S_−15_N_15_, and S_−60_N_60_). In the S_X_N_Y_ notation, X and Y indicate the azimuthal angles (in degrees) of the speech (S) and noise (N) sources with 0° indicating a source directly in front and positive and negative values indicating sources to the right and the left of the midline, respectively.

#### 2.5.3 Stimuli

The full protocol involved measuring 144 SRTs, each of which required passing 20 test sentences (aside from the 10 initial practice sentences). Therefore, the protocol required having access to 2,880 different test sentences to prevent potential confounding effects of sentence repetition from affecting the results. An attempt was made to test all participants using the female sentences in the Spanish version of the Oldenburg Sentence Test, also known as the “matrix” test (Hochmuth et al., 2012) (see Table 2). However, two participants (S4 and S5) could not recognize the matrix sentences, even in quiet and after several opportunities. The two participants in question were tested using the sentences for a male talker of the Castilian Spanish version of the Hearing-in-Noise Test (HINT) (Nilsson et al., 1994; Huarte, 2008) (Table 2).

We deemed it reasonable to use different speech materials for different participants because the aim was to compare performance across processing strategies tested with the same speech material (within-subject comparison design), rather than to compare performance across participants.

Speech reception thresholds were measured for sentences masked by a steady-state speech-shaped noise (SSN) and an international female fluctuating masker (IFFM) (Holube et al., 2010). Different SSN (as provided with the corresponding speech material) was used to mask matrix and HINT sentences to make sure that the SSN spectrum matched the average spectrum of the corresponding sentence material. A different SSN or IFFM token was used to mask each sentence. The masker started 500 ms before the sentence onset and ended 100 ms after the sentence offset and was gated with 50 ms cosine-squared onset and offset ramps. For the IFFM masker, SRTs were measured for speech at −48, −38 and −28 dB FS. For the SSN, the speech level was fixed at −38 dB FS. For reference, the speech level of −38 dB FS corresponds approximately to 65 dB SPL in MED-EL clinical CI audio processors.

#### 2.5.4 Order of testing

Measurements were organized in four blocks:

1. Bilateral and unilateral listening with the speech level at −38 dB FS and with the IFFM masker. This block involved measuring 18 SRTs: 12 SRTs in bilateral listening (3 strategies × 4 spatial configurations) plus 6 SRTs in unilateral listening (3 strategies × 2 spatial configurations).
2. Bilateral listening with speech level at −38 dB FS and with the SSN masker. This block involved measuring 12 SRTs (3 strategies × 4 spatial configurations).
3. Bilateral listening with speech level at −28 dB FS and with the IFFM masker. This block involved measuring 9 SRTs (3 strategies × 3 spatial configurations).
4. Bilateral listening with speech level at −48 dB FS and with the IFFM masker. This block involved measuring 9 SRTs (3 strategies × 3 spatial configurations).

Each block was administered three times, once per SRT estimate. Within each block, test conditions (i.e., target-masker spatial configurations, listening modality, and processing strategies) were administered in random order. Blocks were administered in different orders across participants and repeats. This allowed comparing SRTs across processors and conditions while minimizing potential learning effects. Participants were given brief breaks as needed.

### 2.6 Sound localization

The method was virtually like in our former related publications (e.g., Lopez-Poveda et al., 2019).

#### 2.6.1 Procedure

Participants were presented with acoustic stimuli convolved with HRIRs for each one of 11 azimuthal angles that covered an azimuth range −75° to +75° every 15° (elevation of 0°). During the experiment, participants sat in front of a computer screen. The screen displayed a top view of a human head with a circular array of 37 speakers in front of the head. The speakers covered an azimuth range from −90° to 90° and spaced every 5° (e.g., see Figure 2 in Lopez-Poveda et al., 2019). For each stimulus presentation, the participant was asked to report the perceived azimuthal position of the sound source by clicking on the corresponding speaker in the computer screen. The response triggered the presentation of a new stimulus.

A practice block was administered before the test block. The practice block involved presenting two stimuli per azimuth location in random order (i.e., 22 stimulus presentation in total). The test block involved presenting six stimuli per azimuth location in random order (66 stimulus presentations in total). Several measures were taken to minimize potential learning effects that might have biased scores across strategies. First, the various processing strategies were tested in random order. Second, feedback was not given to participants on the correctness of their responses. Third, participants were blind to the processing strategy they were being tested with (or training on). Lastly, before testing began, participants were encouraged to train themselves on the task. Training involved blindly choosing a processing strategy and listening to stimuli by clicking on each one the 37 speakers of the response screen.

#### 2.6.2 Stimuli

Tests were conducted for two different stimuli:

- Broadband noise bursts: White noise was generated digitally (using MATLAB’s randn function) and bandpass filtered (first-order Butterworth filter) to achieve the desired bandwidth (125–6,000 Hz). The noise bursts had a duration of 500 ms and were gated with 50-ms cosine-squared onset and offset ramps. The mean level was −38 dB FS.
- Sentences in noise: Matrix sentences were presented at a mean level of −38 dB FS. Together with the sentences, a masking SSN was presented at 0 dB SNR and was always located in front of the participant (0° azimuth). The noise started 500 ms before the sentence onset and ended 50 ms after the sentence offset.

To force participants to localize sources based on a “true” interaural level cue rather than on the absolute level at either ear, the actual level for each of the two stimuli used (noise bursts or sentences in noise) was roved randomly by up to ±2 dB across stimulus presentations.

#### 2.6.3 Analysis

Response matrices were produced by plotting the response against the presentation azimuth angles. The RMS localization angle error (*E*_RMS_) was calculated as:


$$E_{\mathrm{RMS}}=\sqrt{\sum_{i=1}^{N}\frac{{\left(A_{i}-R_{i}\right)}^{2}}{N},}$$ (3)

Where *A_i_* and *R_i_* denote the presentation and response azimuth for the *i*-th stimulus, and *N* is the total number of presentations (*N* = 66). Localization accuracy was also assessed using the Pearson correlation coefficient between presentation and response azimuthal angles.

### 2.7 Double-blind approach

All tests were “double blind” such that neither the experimenter nor the participant knew which sound-processing strategy was being tested at any time.

### 2.8 Statistical analysis

SRTs obtained in unilateral and bilateral listening were analyzed separately. The Kolmogorov–Smirnov test (with Lilliefors correction) was used to assess if data were normally distributed. When this happened, two-way repeated-measures analyses of the variance (RMANOVA) were used to test for the statistical significance of processing strategy and spatial configuration on SRTs, and on angle error or correlation scores for the sound localization data. When data were not normally distributed, we applied Friedman and Wilcoxon signed-rank tests instead. An effect was regarded as statistically significant when the null hypotheses could be rejected with 95% confidence (*p* ≤ 0.05). For tests involving multiple groups or variables, *post hoc* pairwise comparisons were conducted using Bonferroni correction of the *p* value for multiple comparisons. All statistical analyses were conducted using IBM SPSS Statistics (v28.0.1.1).

## 3 Results

### 3.1 Example of electrical stimulation patterns

**Figure 3:**
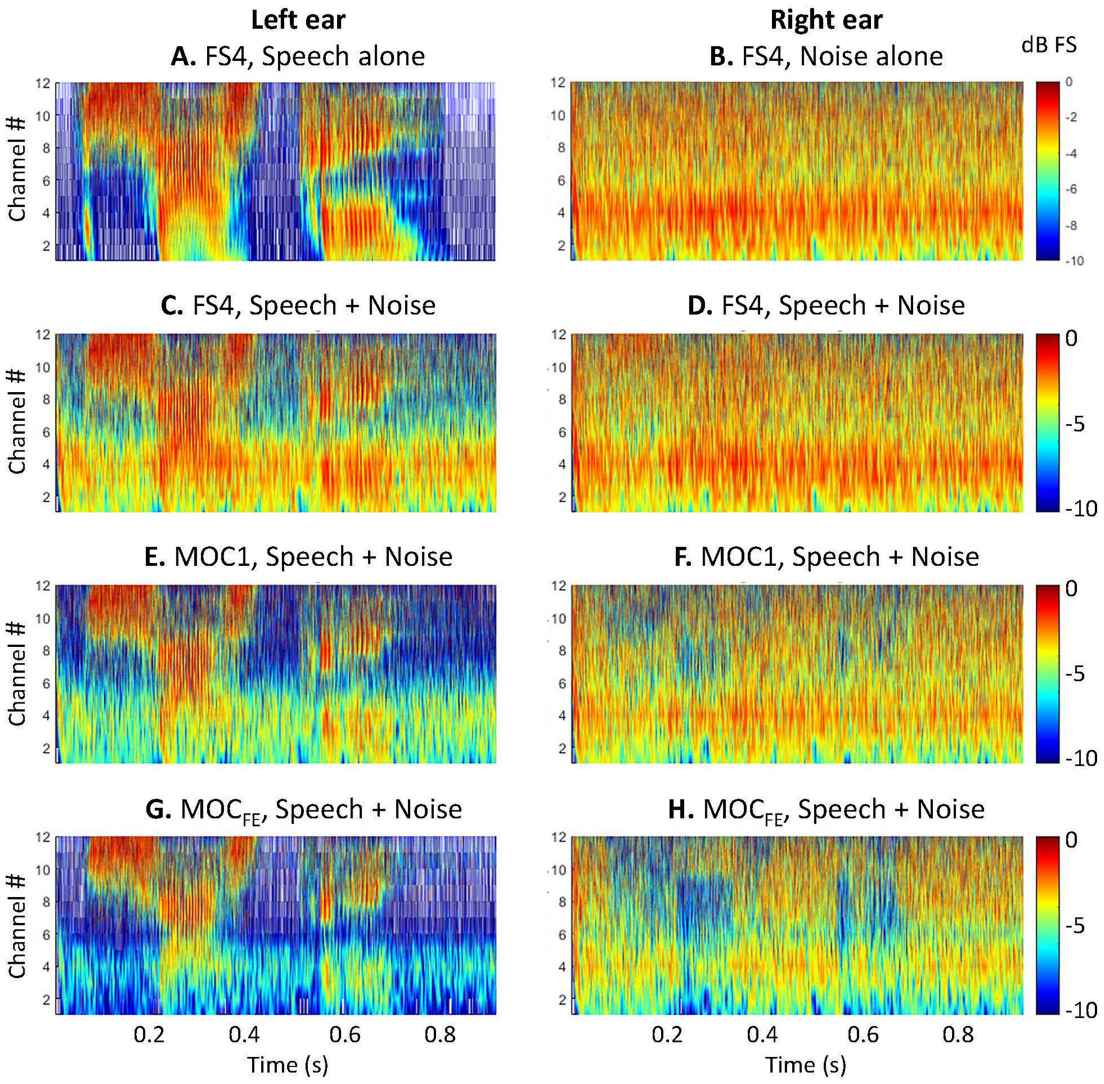
Compressed envelopes at the output of FS4, MOC1 and MOC_FE_ processors with 12 frequency channels. The speech was the Castilian Spanish word “sastre,” and the masker was speech-shaped noise (SSN). The speech and the noise sources were located at −60° and +60° azimuth, respectively. Colour illustrates output amplitude in units of dB FS, and spatial smoothing was applied to improve the view. Each row **(C–H)** is for a different processing strategy, as indicated at the top of each panel. Left and right panels illustrate results for the left- and right-ear processors, respectively. As a reference, the top panels illustrate results for the FS4 strategy for the speech in quiet **(A)** and for the noise alone **(B)**. All other panels illustrate results for the speech and noise at −38 dB FS (0 dB SNR).

To compare the functioning of the FS4, MOC1 and MOC_FE_ strategies, Figure 3 shows the output signals (compressed envelopes) from the three processors. In this example, the stimulus was the disyllabic Spanish word “*sastre*” uttered in simulated free-field conditions by a female speaker located at 300° azimuth (i.e., on the left side of the head) while a noise source was located at 60° azimuth (i.e., on the right side of the head) (S_−60_N_60_ condition). The level of the speech and noise were both −38 dB FS, hence the SNR was 0 dB. As a reference, the top panels illustrate results for the FS4 strategy for the word in quiet (Figure 3A) and for the noise alone (Figure 3B). The figure shows the following:

1. Noise levels were lower for the MOC1 and MOC_FE_ strategies than for the FS4 strategy, particularly in the left ear, the ear closer to the speech source (compare Figures 3C,E,G).
2. In the ear closer to the speech source (the left ear in this example), the MOC1 and the MOC_FE_ strategies provided a better SNR than the FS4 strategy.
3. For the MOC1 and MOC_FE_ strategies, some of the speech features were inhibited in the left ear. The inhibition is greater for the MOC_FE_ than for the MOC1 especially at low frequency channels (channels 1 to 4).
4. In the left ear and in the lower-frequency channels (e.g., channel number 4), noise levels were lower for the MOC1 and MOC_FE_ strategies than for the FS4 strategy. Noise levels were lower for MOC_FE_ than for MOC1.

In summary, overall, the MOC_FE_ strategy behaves like the MOC1 strategy in that they both provide a higher SNR in the ear closer to the speech source than the FS4 strategy. However, the contralateral inhibition is overall stronger for the MOC_FE_ strategy than for the MOC1 strategy.

### 3.2 Speech reception thresholds

#### 3.2.1 SRTs in bilateral listening in fluctuating masker

**Figure 4:**
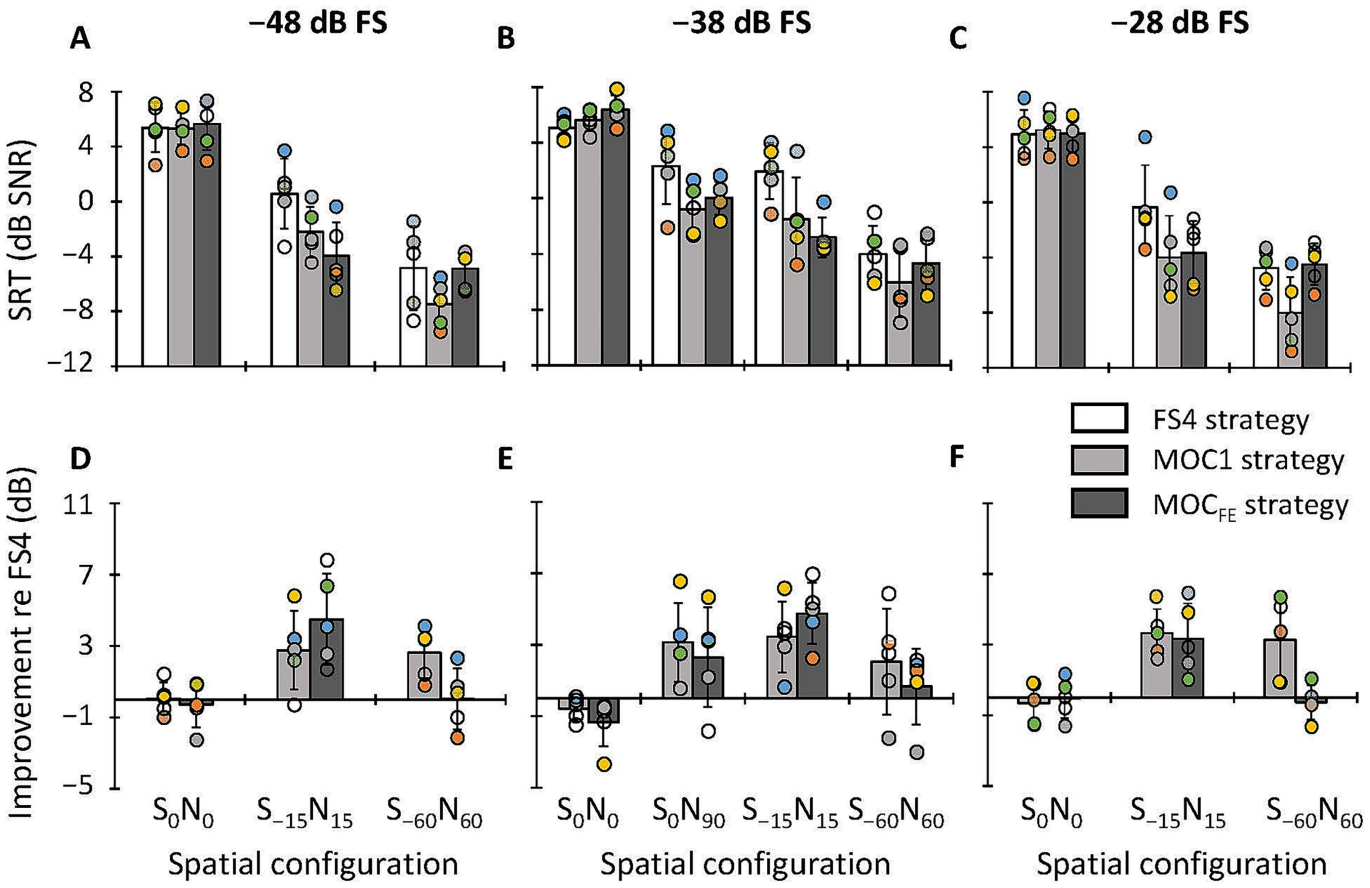
Top **(A–C)** SRTs for sentences in competition with IFFM in bilateral listening with the FS4, MOC1 and MOC_FE_ strategies for speech at −48, −38 and −28 dB FS, respectively. Bottom **(D–F)** SRT improvement (benefit) provided by the tested strategies relative to the FS4 strategy. Circles illustrate individual scores for five bilateral CI users (each individual score is the mean of three SRT estimates), and bars illustrate group mean scores for each processing strategy, as indicated by the inset. In each panel, the abscissa is the spatial configuration of the speech and masker sources. Error bars depict one standard deviation. See the main text for details.

In this section, we compare the SRTs for the MOC1 and MOC_FE_ with those for the FS4 strategy in bilateral listening for speech in IFFM at different speech levels. The top panels in Figure 4 show SRTs for each of the three strategies, as indicated by the inset, for speech at −48, −38 and −28 dB FS, respectively, as indicated at the top of the panel, and for different spatial configurations (S_0_N_0_, S_0_N_90_, S_−15_N_15_, and S_−60_N_60_). Circles illustrate individual data (*N* = 5). Recall that each individual score is the mean of three estimates. Bars show group mean scores. The bottom panels in Figure 4 illustrate SRT improvements (or “benefit” in dB) provided by the MOC1 and MOC_FE_ strategies relative to the reference FS4 strategy. Positive values indicate that SRTs were lower (better) with the test strategy than with the FS4 strategy while negative values indicate that the test strategy was disadvantageous compared to the FS4 strategy. In what follows, results are analyzed separately for the three speech levels.

For speech at −48 dB FS (Figures 4A,D), mean SRTs across participants were equal or better with the MOC1 and MOC_FE_ strategies than with the FS4 strategy for all spatial configurations. A two-way RMANOVA was conducted to test for the effect of processing strategy (FS4, MOC1 and MOC_FE_), spatial configuration (S_0_N_0_, S_−15_N_15_, and S_−60_N_60_), and their interaction on the group mean SRTs. The RMANOVA revealed significant effects of processing strategy [*F*(2,8) = 6.1, *p* = 0.025] and spatial configuration [*F*(2,8) = 148.3, *p* ≤ 0.001], and a significant interaction between strategy and spatial configuration [*F*(4,16) = 13.8, *p* ≤ 0.001]. *Post hoc* pairwise comparisons (with Bonferroni correction) revealed that the mean SRT (across participants and spatial configurations) for any strategy was not significantly different from the mean SRT for any other strategy (the mean SRTs were 0.4, −1.4, and −1.1 dB SNR for FS4, MOC1 and MOC_FE_, respectively). On the other hand, pairwise *post hoc* comparisons revealed that mean SRTs (across strategies and participants) improved with increasing the spatial separation between the speech and noise sources (the mean SRTs were 5.5, −1.9 and −5.7 dB SNR for S_0_N_0_, S_−15_N_15_ and S_−60_N_60_, respectively). *Post hoc* pairwise comparisons revealed that SRTs were statistically better for spatial configurations where the speech and noise sources were spatially separated (S_0_N_0_ versus S_−15_N_15_, *p* = 0.002; S_0_N_0_ versus S_−60_N_60_, *p* ≤ 0.001; and S_−15_N_15_ versus S_−60_N_60_, *p* = 0.017). In addition, a *post hoc* analysis of the interaction between spatial configuration and processing strategy showed that the three strategies produced better SRTs when the target and the masker were at separate locations, i.e., produced significant spatial release from masking (FS4 strategy: S_0_N_0_ versus S_−15_N_15_, *p* = 0.002; S_0_N_0_ versus S_−60_N_60_, *p* = 0.001; S_−15_N_15_ versus S_−60_N_60_, *p* = 0.010; MOC1 strategy: S_0_N_0_ versus S_−15_N_15_, *p* = 0.007; S_0_N_0_ versus S_−60_N_60_, *p* ≤ 0.001; S_−15_N_15_ versus S_−60_N_60_, *p* = 0.014; and MOC_FE_ strategy: S_0_N_0_ versus S_−15_N_15_, *p* = 0.001; S_0_N_0_ versus S_−60_N_60_, *p* ≤ 0.001; S_−15_N_15_ versus S_−60_N_60_, not significant). The interaction between processing strategy and spatial configuration showed that for S_−15_N_15_ spatial configuration, SRTs tended to be higher (worse) for the FS4 than for the MOC_FE_ strategy (*p* = 0.052). For S_−60_N_60_ spatial configuration, SRTs were higher (worse) for the FS4 (*p* = 0.045) and MOC_FE_ (*p* ≤ 0.001) strategies than for the MOC1 strategy. For the S_0_N_0_ spatial configuration, the effect of strategy on SRTs was not significant.

For speech at −38 dB FS (Figures 4B,E), the MOC1 and MOC_FE_ strategies improved SRTs for all spatial configurations except for the collocated condition (S_0_N_0_), where SRTs tended to be slightly better with the FS4 strategy. The RMANOVA revealed significant main effects of processing strategy [*F*(2,8) = 13.5, *p* = 0.003], spatial configuration [*F*(3,12) = 114.7, *p* ≤ 0.001] and a significant interaction between strategy and spatial configuration [*F*(6,24) = 5.2, *p* = 0.001] on the group mean SRTs. *Post hoc* pairwise comparisons (with Bonferroni correction) revealed that the group mean SRT was significantly better with the MOC1 than with the FS4 strategy (−0.7 versus 1.3 dB SNR, respectively, *p* = 0.024). In addition, the mean SRT tended to be better for the MOC_FE_ than for the FS4 strategy, but this effect was not statistically significant (−0.3 versus 1.3 dB SNR, respectively, *p* = 0.077). The SRTs for the MOC1 and MOC_FE_ strategies were not significantly different from each other (−0.7 versus −0.3 dB SNR, respectively, *p* = 0.903). On the other hand, the mean SRTs (across strategies and participants) improved with increasing the spatial separation between the speech and noise sources (mean SRTs were 5.7, 0.5, −0.8 and −4.9 dB SNR for S_0_N_0_, S_0_N_90_, S_−15_N_15_ and S_−60_N_60_, respectively). *Post hoc* pairwise comparisons revealed significant differences in SRTs across some spatial configurations (S_0_N_0_ versus S_0_N_90_, *p* = 0.002; S_0_N_0_ versus S_−15_N_15_, *p* = 0.004; S_0_N_0_ versus S_−60_N_60_, *p* ≤ 0.001; S_0_N_90_ versus S_−60_N_60_, *p* = 0.004; and S_−15_N_15_ versus S_−60_N_60_, *p* = 0.018). In addition, they revealed that the tested processing strategies produced significant spatial release from masking [FS4 strategy (S_0_N_0_ versus S_−60_N_60_, *p* = 0.001; S_0_N_90_ versus S_−60_N_60_, *p* = 0.050; S_−15_N_15_ versus S_−60_N_60_, *p* = 0.031); MOC1 strategy (S_0_N_0_ versus S_0_N_90_, *p* = 0.002; S_0_N_0_ versus S_−15_N_15_, *p* = 0.017; S_0_N_0_ versus S_−60_N_60_); MOC_FE_ strategy (S_0_N_0_ versus S_0_N_90_, *p* = 0.010; S_0_N_0_ versus S_−15_N_15_, *p* = 0.002; S_0_N_0_ versus S_−60_N_60_, *p* = 0.003; S_0_N_90_ versus S_−15_N_15_, *p* = 0.018; S_0_N_90_ versus S_−60_N_60_, *p* = 0.002)]. In addition, *post hoc* comparisons of the interaction between strategy and spatial configuration showed that for S_−15_N_15_ spatial configuration, the mean SRT was higher (worse) for the FS4 strategy than for the MOC_FE_ strategy (*p* = 0.010) and tended to be worse for the FS4 than for the MOC1 strategy (*p* = 0.054). For the other spatial configurations tested, the effect of strategy on SRTs was not significant.

For speech at −28 dB FS (Figures 4C,F), the overall mean SRT was equal or better with the MOC1 and MOC_FE_ strategies than with the FS4 strategy for all spatial configurations tested. The Kolmogorov–Smirnov test (with Lilliefors correction) revealed that the SRTs across processing strategies, spatial configurations and participants did not conform to a normal distribution (*p* = 0.013). The Friedman test revealed that SRTs were statistically different across the nine test conditions (3 processing strategies × 3 spatial configurations) [*χ*^2^(8) = 35.4; *p* ≤ 0.001]. A *post hoc* pairwise analysis with the Wilcoxon signed rank test with Bonferroni correction for multiple comparisons showed that the SRT was significantly better with the MOC1 than with the FS4 strategy (−2.3 versus −0.1 dB SNR, *Z* = −2.6; *p* = 0.027). However, the mean SRT was not significantly different between the FS4 and MOC_FE_ strategies (−0.1 versus −1.1 dB SNR, *Z* = −1.5; *p* = 0.375), or between the MOC1 and MOC_FE_ strategies (−2.3 versus −1.1 dB SNR, *Z* = −1.7; *p* = 0.264). In addition, the mean SRT (across strategies and participants) improved with increasing the spatial separation between the speech and noise sources (mean SRTs were 5.0, −2.7 and −5.8 dB SNR for S_0_N_0_, S_−15_N_15_ and S_−60_N_60_, respectively). A pairwise *post hoc* analysis revealed that the SRTs were statistically better for spatial configurations where the speech and noise sources were spatially separated (S_0_N_0_ versus S_−15_N_15_, *Z* = −3.4; *p* ≤ 0.001; S_0_N_0_ versus S_−60_N_60_, *Z* = −3.4; *p* ≤ 0.001; S_−15_N_15_ versus S_−60_N_60_, *Z* = −3.1; *p* = 0.006). In addition, *post hoc* comparisons showed no significant interaction between processing strategy and spatial configuration (*p* > 0.05).

#### 3.2.2 SRTs in bilateral listening in steady noise

**Figure 5:**
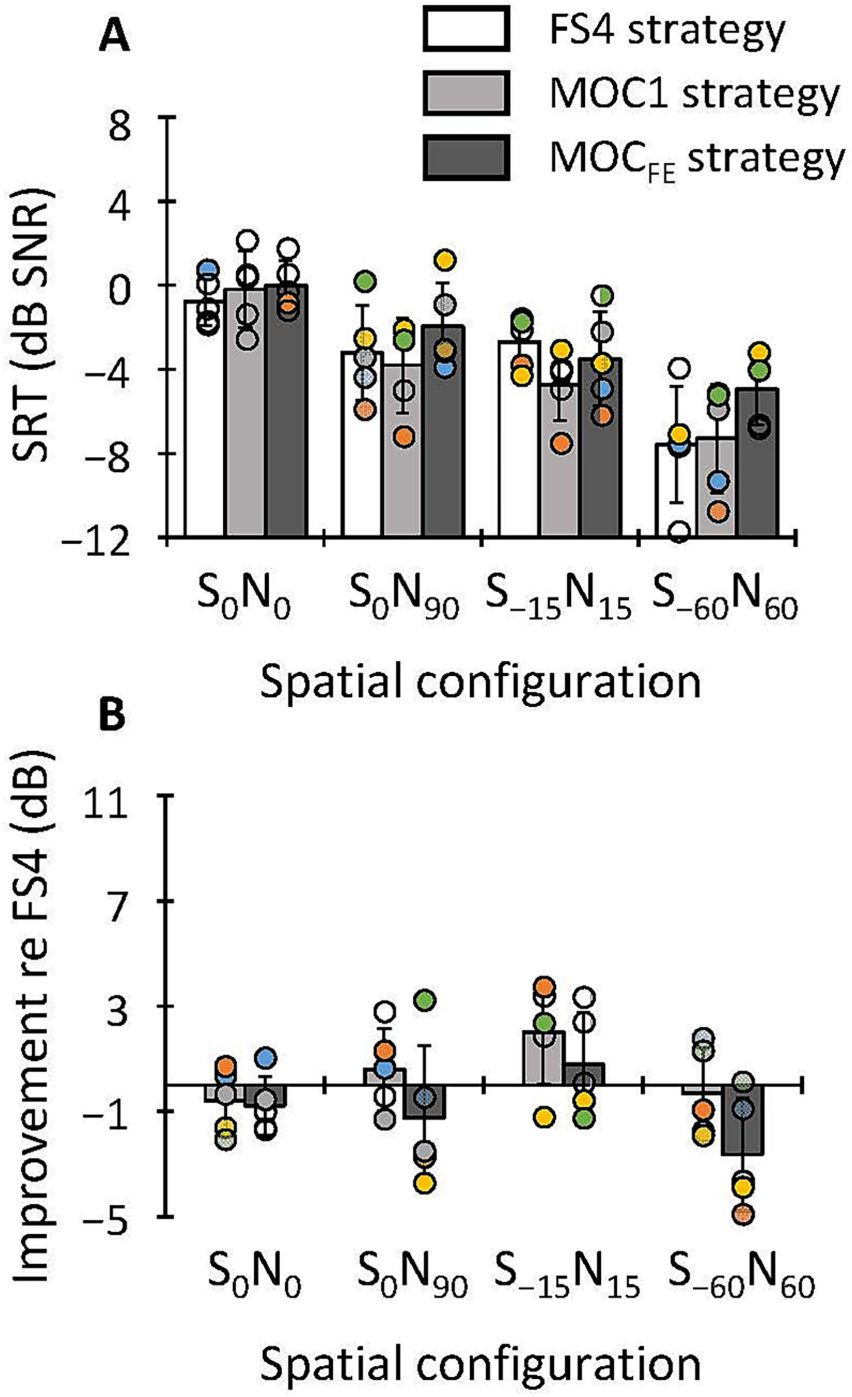
**(A)** SRTs for sentences in competition with SSN in bilateral listening with the FS4, MOC1 and MOC_FE_ strategies for speech at −38 dB FS, and for four spatial configurations of the speech and noise sources, as indicated by the abscissa. **(B)** SRT improvement (benefit) provided by the tested strategies relative to the FS4 strategy. Error bars depict one standard deviation.

Figure 5 illustrates the SRTs for speech in competition with SSN. SRTs were measured in bilateral listening for speech at −38 dB FS, and for S_0_N_0_, S_0_N_90_, S_−15_N_15_ and S_−60_N_60_ spatial configurations. The MOC1 and MOC_FE_ strategies produced similar or worse SRTs than the FS4 strategy for all spatial configurations, except for the S_−15_N_15_ spatial configuration, where these two processing strategies produced better SRTs. A two-way RMANOVA revealed a significant effect of spatial configuration [*F*(3,12) = 39.7, *p* < 0.001] and a significant interaction between strategy and spatial configuration [*F*(6,24) = 3.1, *p* = 0.022] on the group mean SRTs. However, the effect of processing strategy was not statistically significant [*F*(2,8) = 3.9, *p* = 0.064]. A pairwise *post hoc* analysis with Bonferroni correction for multiple comparisons revealed that SRTs were better with increasing the spatial separation of the speech and noise sources (mean SRTs across participants and strategies were −0.3, −2.9, −3.6 and −6.6 dB SNR for S_0_N_0_, S_0_N_90_, S_−15_N_15_ and S_−60_N_60_, respectively). In addition, we found significant differences in mean SRTs (pooled across participants and processing strategies) between some spatial configurations (S_0_N_0_ versus S_−15_N_15_, *p* = 0.017; S_0_N_0_ versus S_−60_N_60_, *p* = 0.007; S_0_N_90_ versus S_−60_N_60_, *p* = 0.002; S_−15_N_15_ versus S_−60_N_60_, *p* = 0.023).

#### 3.2.3 SRTs in unilateral listening in fluctuating masker

**Figure 6:**
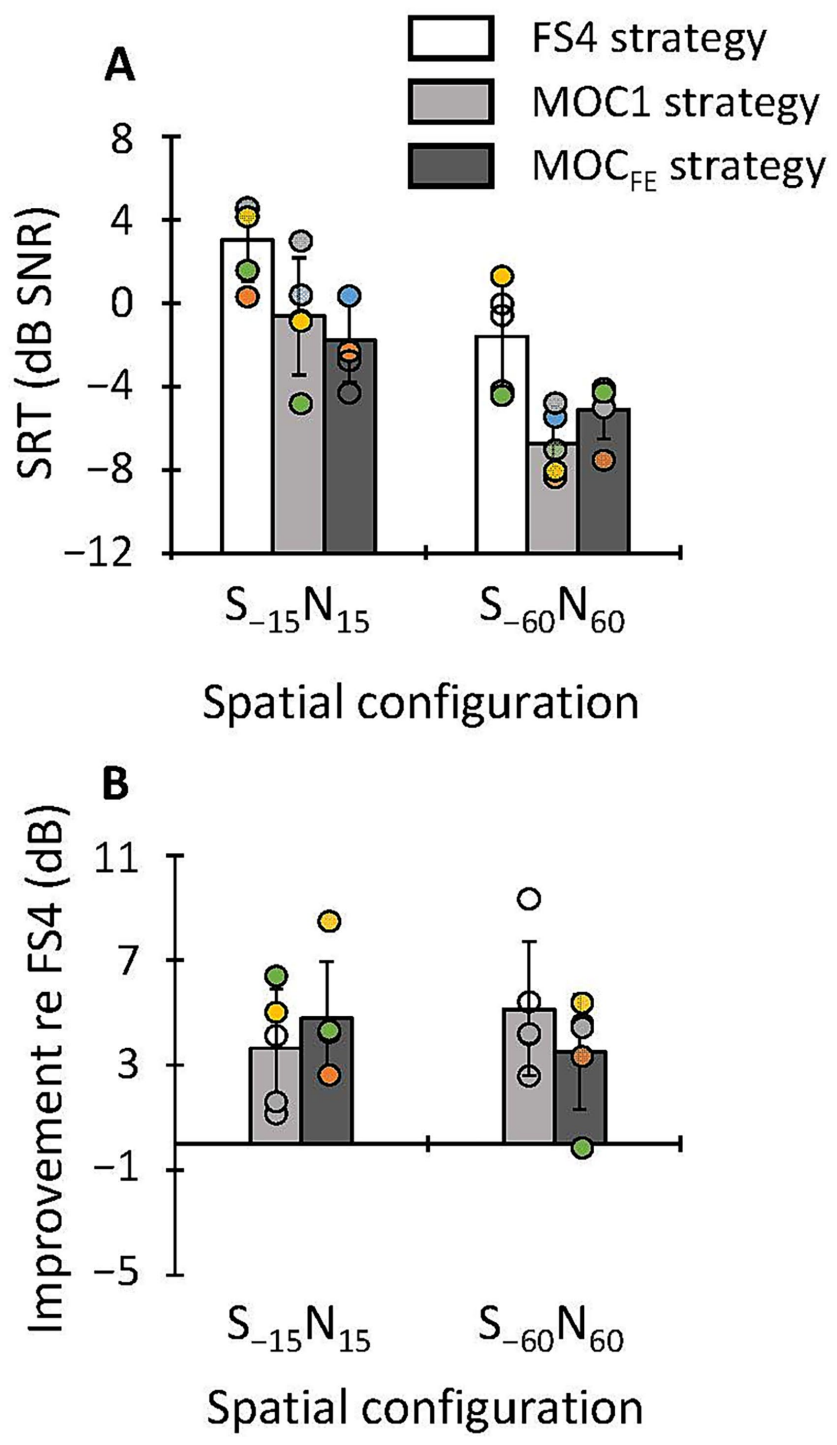
**(A)** SRTs for sentences in competition with IFFM in unilateral listening with the FS4, MOC1 and MOC_FE_ strategies for speech at −38 dB FS, and for two spatial configurations of the speech and noise sources, as indicated by the abscissa. **(B)** SRT improvement (benefit) provided by the tested strategies relative to the FS4 strategy. Error bars depict one standard deviation.

In unilateral listening, SRTs were measured for speech at −38 dB FS in competition with IFFM, and for S_−15_N_15_ and S_−60_N_60_ spatial configurations. Results are shown in Figure 6. On average, SRTs were better with the MOC1 and MOC_FE_ strategies than with the FS4 strategy for the two spatial configurations tested. The RMANOVA revealed a significant main effect of processing strategy [*F*(2,8) = 21.2, *p* < 0.001] and spatial configuration [*F*(1,4) = 59.4, *p* = 0.002] on the group mean SRTs. The interaction between processing strategy and spatial configuration was not significant [*F*(2,8) = 2.5, *p* = 0.144]. *Post hoc* pairwise comparisons with Bonferroni correction revealed that the mean SRTs (across spatial configurations and participants) were significantly better with the MOC1 and the MOC_FE_ than with the FS4 strategy (−3.7 versus 0.7 dB SNR, *p* = 0.017; and −3.4 versus 0.7 dB SNR, *p* = 0.022, respectively). The SRTs with the MOC1 and MOC_FE_ strategies were not significantly different from each other (*p* = 1.0). In addition, *post hoc* pairwise comparisons revealed that SRTs were significantly better for the S_−60_N_60_ than for S_−15_N_15_ spatial configuration (−4.5 versus 0.2 dB SNR, *p* = 0.002).

### 3.3 Sound localization

**Figure 7:**
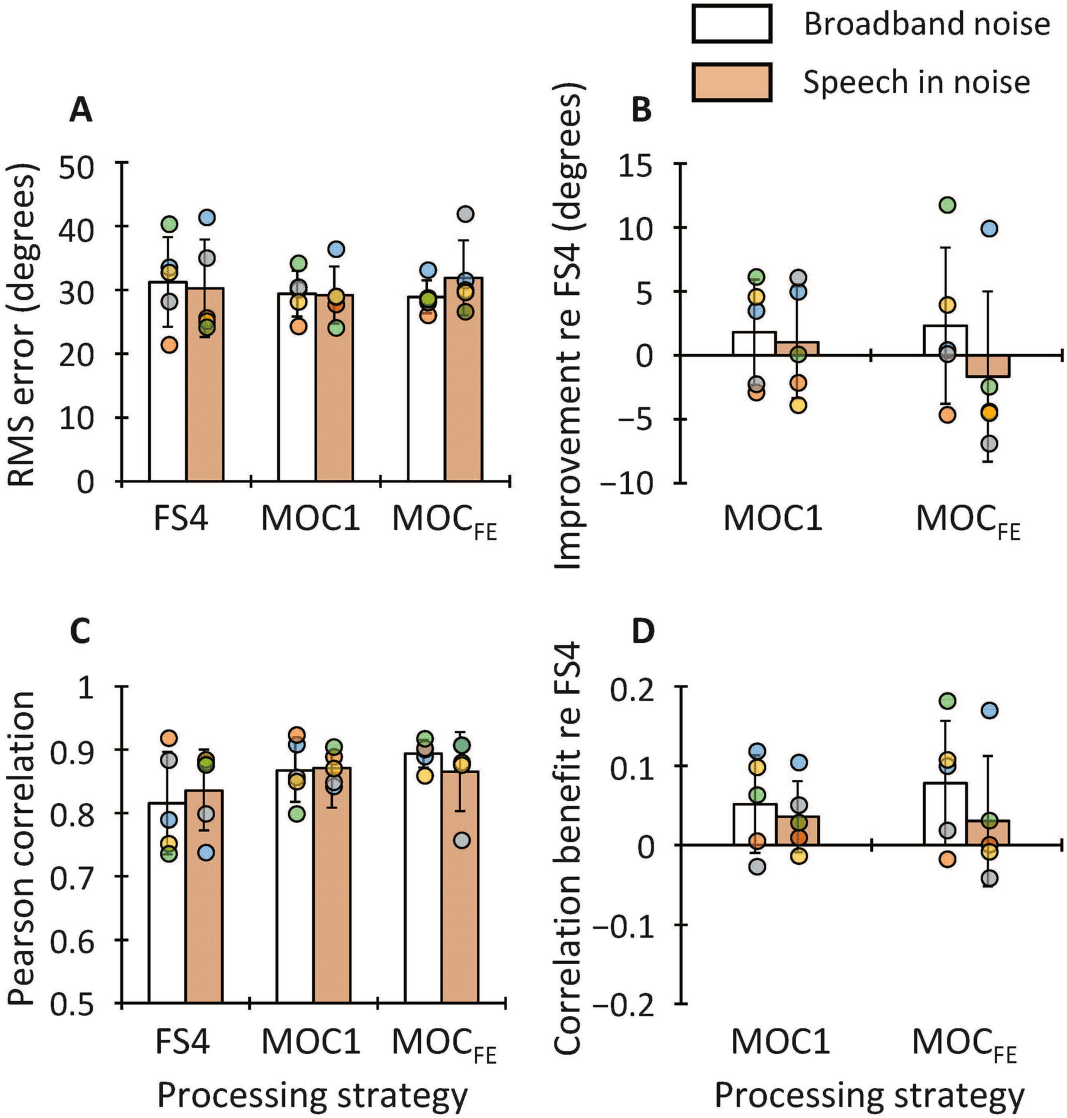
**(A)** Angle error for the FS4, MOC1 and MOC_FE_ strategies, as indicated by the abscissa. Results are shown for each individual participant (circles) and the mean across participants (bars). White and orange bars illustrate results for broadband noise and speech in noise, as indicated by the inset. Lower values indicate better performance. **(B)** Angle error improvement provided by the MOC1 and MOC_FE_ strategies relative to the FS4 strategy. Positive values indicate better performance. **(C)** Correlation between presentation and response azimuth for the FS4, MOC1 and MOC_FE_ strategies. Note that the ordinate scale starts at 0.5 rather than 0.0 to better show small differences. **(D)** Correlation improvement provided by the MOC1 and MOC_FE_ strategies relative to the FS4 strategy. Error bars depict one standard deviation.

Figure 7A illustrates individual and group mean localization angle error scores (Equation 3) for the FS4, MOC1 and MOC_FE_ strategies and the improvement angle error of the MOC1 and MOC_FE_ strategies relative to the FS4 strategy (Figure 7B) for each individual participant (circles) and the mean across participants (bars), when the stimulus was a broadband noise (white bars) or a sentence in noise (shaded bars). When the stimulus was a broadband noise, the mean angle error tended to be equal or better (smaller) with the MOC1 and MOC_FE_ strategies (mean ± S.D. = 29.4° ± 3.6° and 28.9° ± 2.6°, respectively) than with the FS4 strategy (31.2° ± 7.0°). When the stimulus was sentences in noise, the mean angle error tended to be better with the MOC1 strategy (mean ± S.D. = 29.2° ± 4.5°) than with the MOC_FE_ or the FS4 strategies (31.9° ± 5.9° and 30.2° ± 7.6°, respectively). The RMANOVA revealed that the effect of processing strategy [*F*(2,8) = 0.5, *p* = 0.633], stimulus type [*F*(1,4) = 0.04, *p* = 0.843] and the interaction between processing strategy and stimulus type [*F*(2,8) = 0.6, *p* = 0.548] were not statistically significant on group mean localization angle error scores.

Figure 7C depicts the Pearson correlation coefficient between the actual stimulus presentation and response angles for the FS4, MOC1 and MOC_FE_ strategies, and the improvement (or benefit) of the MOC1 and MOC_FE_ strategies relative to the FS4 strategy (Figure 7D). On average, independently of the stimulus, the correlation tended to be higher (better) with the MOC1 and MOC_FE_ strategies (mean ± S.D. combining the two stimuli = 0.87 ± 0.04 and 0.88 ± 0.05, respectively) than with the FS4 strategy (0.83 ± 0.07). Kolmogorov–Smirnov tests (with Lilliefors correction) revealed that the correlation coefficient scores did not conform to a normal distribution (*p* = 0.007). A Friedman test revealed that the correlation between the actual and reported azimuth was not statistically different across the processing strategies and the stimuli used (3 processing strategies × 2 stimuli) [*χ*^2^(5) = 3.9; *p* = 0.554].

## 4 Discussion

The aim of the present study was to present and experimentally evaluate a version of the binaural MOC strategy designed to operate in the frequency domain and at the front end rather than the back end of processing. The evaluation involved comparing the performance of five CI users on several hearing tests with the MOC_FE_ as well as with two reference strategies: a backend MOC strategy (MOC1) and a standard FS4 strategy that involved using two independently functioning devices with fixed backend compression. The evaluation tests included (1) speech-in-noise recognition in unilateral and bilateral listening, for different speech levels (−48, −38 and −28 dB FS), for speech in competition with two different maskers (IFFM and SSN), and for multiple spatial configurations of the speech and noise sources (S_0_N_0_, S_0_N_90_, S_−15_N_15_, and S_−60_N_60_); and (2) localization of sound sources in quiet and in competition with a noise source.

### 4.1 Speech reception thresholds

#### 4.1.1 SRTs in bilateral listening at different spatial configurations and speech levels in fluctuating masker

On average, the MOC1 and MOC_FE_ strategies tended to improve SRTs in all spatial configurations except in the collocated condition (S_0_N_0_), where SRTs were approximately equal or slightly better with the FS4 strategy (Figure 4). This finding is consistent with our earlier studies (Lopez-Poveda et al., 2016a; Lopez-Poveda and Eustaquio-Martín, 2018; Lopez-Poveda et al., 2020). The lack of a benefit of MOC processing for the S_0_N_0_ condition could be because when stimuli are identical at the two ears the mutual inhibition between the pair of MOC processors decreased the overall stimulation and reduced the audibility and/or because it distorted the envelopes. Note that the mutual inhibition would be less problematic in realistic listening conditions because any asymmetry in the placement of the CI microphones would cause the levels of the input signals to be different at the two ears.

In addition, speech-in-noise intelligibility benefits provided by the MOC_FE_ strategy (relative to the FS4 strategy) were similar to those provided by the MOC1 strategy for the S_0_N_90_, S_−15_N_15_ spatial configurations. However, for the S_−60_N_60_ configuration the MOC_FE_ strategy showed smaller benefits than the MOC1 strategy (Figures 4D–F). The latter may have occurred because the magnitude of contralateral inhibition in the S_−60_N_60_ condition was so large with MOC_FE_ strategy that it decreased the overall stimulation level (see Figure 3G) and hindered speech recognition due to compromised audibility. The issue might be solved by adjusting the inhibition parameters in the MOC_FE_ strategy.

Overall, SRTs were equal or better with the MOC1 and MOC_FE_ strategies than with the FS4 strategy at the three speech levels tested. The benefit from the tested strategies tended to be greater for speech at −38 dB FS than at −48 or −28 dB FS (compare the bottom panels in Figure 4). This finding is consistent with the data shown in a previous study with a backend MOC strategy with slower (*τ_a_* = 2 ms, *τ_b_* = 300 ms) contralateral inhibition, namely the MOC3-FS4 strategy (Fumero et al., 2021), and suggests that the MOC1 and MOC_FE_ strategies provide a benefit for speech levels of about 65 dB SPL and could also provide some benefits for speech of 55 and 75 dB SPL.

#### 4.1.2 SRTs in bilateral listening with different masker types

Compared to the FS4 strategy, the MOC1 and MOC_FE_ strategies improved SRTs for sentences in fluctuating noise (mean SRT improvement of 2.0 and 1.6 dB SNR, respectively), but did not improve or degrade SRTs for sentences in stationary noise (mean SRT improvement of 0.4 and −1.0 dB SNR, respectively) when tests were conducted at −38 dB FS (compare Figures 4B,E with Figures 5A,B). This finding is consistent with a previous study where we showed that the MOC1 strategy improves speech recognition in competition with a single-talker masker (Lopez-Poveda et al., 2017). However, it differs from other studies where we observed that backend MOC processing improved speech recognition also in stationary noise (Lopez-Poveda and Eustaquio-Martín, 2018; Lopez-Poveda et al., 2020; Fumero et al., 2021). The discrepancy may be due to differences in the time course of MOC processing. Here, we have used a MOC_FE_ implementation that involves fast contralateral control of compression that enhances the SNR in the ear contralateral to the masker (ipsilateral to the speech) when the masker is fluctuating (see Figure 1 in Lopez-Poveda et al., 2020). However, in stationary noise, the benefits of MOC processing, at least for the backend implementation, seem to be larger with longer time constants (see, e.g., Lopez-Poveda and Eustaquio-Martín, 2018) because the fast fluctuations of the stationary noise combined with fast contralateral controlled compression could cause distortion. This suggests that the greater benefit for fluctuating than for stationary noise is likely due to the use of fast time constants of binaural MOC control, and that using a longer time constant (e.g., 300 ms instead of 2 ms) could increase the benefit of the MOC_FE_ in stationary noise (see the related discussion on p. 1507 of Lopez-Poveda et al., 2020). The issue deserves further investigation.

#### 4.1.3 SRTs in unilateral listening in fluctuating masker

In unilateral listening, SRTs were significantly better with the MOC1 and MOC_FE_ strategies (mean SRT improvement of 4.4 and 4.1 dB SNR, respectively), than with the FS4 strategy for the two spatially separated configurations tested (Figure 6). This is consistent with our previous studies where we showed that the MOC1 strategy was advantageous over two independently functioning CIs in unilateral listening when the implanted ear had the better acoustic SNR (stationary noise: Lopez-Poveda et al., 2016a, 2020; and single-talker maskers: Lopez-Poveda et al., 2017).

In addition, for the S_−60_N_60_ spatial configuration, the MOC1 and MOC_FE_ strategies tended to improve SRTs (relative to the FS4 strategy) more in unilateral listening than in bilateral listening (compare Figure 6B with Figure 4E). The MOC1 strategy improved the mean SRT by 5.1 dB SNR in unilateral listening versus 2.0 dB SNR in bilateral listening, while with the MOC_FE_ strategy, the corresponding improvements were 3.5 versus 0.7 dB SNR, respectively. The reason for this trend toward better recognition in unilateral listening is not clear. Unilateral listening tests were conducted at the same time as bilateral listening tests, so participants were equally accustomed to MOC processing at the time when bilateral and unilateral listening tests were performed. A possible explanation for the greater benefit of MOC processing (relative to the FS4 strategy) in unilateral than in bilateral listening in this specific spatial configuration (S_−60_N_60_) could be that MOC processing improves the SNR in the ear with the better acoustic SNR (i.e., the tested ear in unilateral listening condition), while it decreases the SNR in the contralateral ear (Lopez-Poveda and Eustaquio-Martín, 2018 and in Figure 3 in Lopez-Poveda et al., 2020) and, perhaps, the SNR decrease in the contralateral ear causes some form of bilateral interference that reduces the benefit from MOC processing.

#### 4.1.4 Spatial release from masking

SRTs improved by increasing the spatial separation between the target and masker sources. This improvement, known as spatial release from masking (Litovsky, 2012), occurred for all strategies (FS4, MOC1 and MOC_FE_), speech levels, listening modes, and masker types (Figures 4–6). The mean spatial release from masking in bilateral listening (across all stimulus presentation levels and masker types) for the S_−15_N_15_ versus the S_0_N_0_ condition was largest for the MOC_FE_ (7.7 dB), slightly smaller for the MOC1 strategy (7.1 dB), and smallest for the FS4 (3.8 dB) strategy. In addition, for the S_−60_N_60_ versus S_0_N_0_ conditions, the mean spatial release from masking was largest for the MOC1 (11.2 dB), midrange for the MOC_FE_ strategy (9.0 dB), and smallest for the FS4 (8.9 dB) strategy (Figures 4, 5). In unilateral listening (Figure 6), the spatial release from masking for the S_−60_N_60_ versus S_−15_N_15_ conditions was largest for the MOC1 (6.1 dB), midrange for the FS4 (4.6 dB), and smallest for the MOC_FE_ (3.3 dB) strategies. These results demonstrate that the MOC_FE_ strategy provides as much spatial release from masking as backend MOC processing (Lopez-Poveda et al., 2016a; Lopez-Poveda et al., 2017; Lopez-Poveda et al., 2020). In addition, these outcomes are broadly consistent with those reported by Loizou et al. (2009) and Gifford et al. (2014) in bilateral CI users, where they observed an overall spatial release from masking of 2–5 dB and 3–5 dB, respectively.

### 4.2 Sound source localization

We have shown that, compared to using two independently functioning FS4 processors, the MOC1 and MOC_FE_ strategies tended to improve the localization (mean angle error = 31.2°, 29.4° and 28.9°, respectively) of a broadband noise in a virtual horizontal plane (Figure 7A). These scores are close to or higher (worse) than those reported in a previous study (25.3° with two independently functioning CIs versus 22.7° with the MOC1 strategy; Lopez-Poveda et al., 2019). The slightly higher errors found in the present study could be due to differences in the implementation of the strategies. Specifically, in the previous study, the reference strategy was a continuous interleaved sampling (CIS) strategy (Wilson et al., 1991) rather than FS4, and both the reference and the MOC1 strategies were implemented without linked AGC. The participants of the present study did not use linked AGC in their clinical CIs and were not given time to acclimatize to the linked AGC. Therefore, the linked AGC could have provided participants with new or distorted sound localisation cues (e.g., interaural level differences).

When the stimulus consisted of sentences in noise, the mean angle error scores were broadly similar for the three strategies (MOC1, 29.2°; MOC_FE_, 31.9°; and FS4, 30.2°; Figure 7B). In addition, the mean angle localization errors for the speech stimulus were similar to those reported in previous studies where CIs users were tested on a similar task in a free field environment (22° in Verschuur et al., 2005; 21.5° in Grantham et al., 2007; 42° in Neuman et al., 2007). These outcomes suggest that the implementation of MOC_FE_ holds promise for improving speech in noise intelligibility without altering sound source localization accuracy.

Previous studies have reported that CI users localize speech stimuli significantly better than nonspeech stimuli (Verschuur et al., 2005; Grantham et al., 2007). In contrast with this, we found no differences in localization accuracy for broadband noise and sentences in noise (with the reference FS4 strategy, the mean angle error was approximately 30° for the two types of stimuli; Figure 7A). This result might be attributable to the fact that the task in the present study was more challenging because the speech source was always presented in competition with a noise source in front of the participant (0° azimuth) rather than in quiet.

Although the correlation between the presentation and the response azimuth was not statistically different across the processing strategies (Figure 7C), the correlation tended to be higher (better) with the MOC1 and MOC_FE_ strategies than with the FS4 strategy for the two stimuli used (Figure 7D). This indicates that, for the two stimuli used, participants tended to localize sounds more accurately with the MOC1 and MOC_FE_ strategies than with the FS4 strategy even though the latter was most similar to their clinical audio processor. This suggests that localization with the MOC1 and MOC_FE_ strategy could improve with practice and/or sustained use of these strategies.

### 4.3 Limitations and practical implementation issues

In the present study, stimuli were processed via software simulations of the processing strategies and were presented directly to the listener via a research interface. Free-field target-masker spatial configurations were simulated by convolving monophonic sound recordings through appropriate HRIRs resulting in static heads and sound sources. The employed HRIRs were for the behind-the-ear MED-EL SONNET sound processor placed on an artificial head and torso and were recorded in a nearly anechoic chamber. In addition, target and masker were limited to a single source of each at specific locations. In clear contrast with this, in realistic listening scenarios, CI users hear through a pair of wearable devices, spatial cues are provided by their own HRIRs, sound sources are mobile (dynamic) and arrive from different distances and directions around the listener, and reverberation is often present (Weisser et al., 2019). Furthermore, the type and location of the target and maskers may vary from moment to moment which requires head movements for the listener to localize them (Mansour et al., 2021). These factors were not considered in the current evaluations and may affect the advantages of the MOC_FE_ strategy.

The present evaluations were limited to a specific implementation of the MOC_FE_ strategy with a specific set of parameters. Implementing the MOC_FE_ strategy in audio processor devices would require exchanging data (contralateral output levels) bilaterally, from ear to ear. We chose to evaluate this strategy with four spectral bands and fast contralateral control of expansion (*τ_a_* = *τ_b_* = 2 ms) because two speech intelligibility models (the short-term objective intelligibility, Taal et al., 2011; and the output signal-to-noise ratio, Watkins et al., 2018) predicted intelligibility improvements with minimal bilateral exchange of data, and acceptable contralateral inhibition (<3 dB). However, the benefits might be greater with different implementations and/or parameters.

Implementing the MOC_FE_ strategy in actual devices would require exchanging data between the pair of CIs. In the present study, this exchange was assumed to be without delays. An implementation of the MOC_FE_ in actual devices would probably involve using a wireless communication protocol, which would almost certainly introduce delays. It remains uncertain to what extent those delays may affect the benefit provided by the MOC_FE_, but it may be necessary to review the strategy and/or the parameters considering the potential delays.

In the present study, the three strategies (FS4, MOC1, and MOC_FE_) were implemented and tested using an equal number of active electrodes on the two CIs and, correspondingly, an equal number of filters in the filter banks. This was intended to allow a fair comparison between strategies, i.e., so that any difference in patient performance with the three strategies was due to the use of dynamic compression/expansion and not to other factors such as the number of active electrodes. However, only the MOC1 strategy needs to have the same number of bands and active electrodes in the left and right ears. Indeed, one advantage of the MOC_FE_ over the (backend) MOC1 is that in the MOC_FE_ the number of bands in the FFT (Figure 1) can be set independently from the number of active electrodes.

At the time of conducting the reported evaluations, all participants had a long daily experience with an audio-coding strategy similar to the FS4 strategy (Table 1). In contrast, their experience with the MOC_FE_ strategy was limited to a few days during the evaluation sessions. Because of this, the found benefits of MOC_FE_, although small, are promising. For CI users, the time of use and training can produce significant improvements in speech recognition (e.g., Dorman and Spahr, 2006). Therefore, it is conceivable that the benefits from the MOC_FE_ strategy could become larger with more practice and/or an acclimatization period with this strategy.

Additional research is needed to further explore the potential benefits of MOC_FE_ with other parameters, and in realistic and challenging acoustic situations through a hardware implementation of this strategy.

### 4.4 Generalizability of the concept and other applications

The MOC_FE_ was conceptualized as a version of the backend MOC strategy that could be implemented in the audio domain and at the preprocessing stage of CI processing (Figure 1). The implementation shown and tested here was specifically designed for MED-EL CIs. Because of this, the expansion function (Equation 2) was the inverse of the compression implemented in MED-EL CIs (Equation 1). However, since all CIs use fixed compression to map the broad dynamic range of acoustic hearing to the narrower dynamic range of electric hearing (Wouters et al., 2015), the MOC_FE_ concept (i.e., the use of a contralaterally controlled dynamic expansion at the front end of processing) may be generalized to any pair of CIs. Indeed, the MOC_FE_ concept may be generalized to any pair of hearing devices that use (fixed) compression. The expansion function, however, may need to be adjusted to the specific compression used by the devices in question.

The idea of preprocessing stimuli using a dynamic (time-varying) expansion function may be generalized to applications beyond improving hearing devices. For example, the characteristics of human cochlear compression are still not fully understood. Since otoacoustic emissions (OAEs) are a byproduct of cochlear compression, it may be possible to elucidate the characteristics of human compression by preprocessing OAE stimuli through an expansion function before presenting them to the ear. By doing so, it may be possible to elucidate cochlear compression as the inverse of the expansion function that minimizes, or cancels, OAEs.

Lastly, the concept of binaural inhibition is gaining attraction in auditory modeling, ranging from more detailed models of the MOCR (Smalt et al., 2014) to simpler binaural inhibition stages used in current binaural loudness models (e.g., Moore et al., 2014; Pieper et al., 2021). Placing a dynamic, contralaterally controlled expansion at the front end of processing might be an alternative way to mimic MOCR effects in auditory models.

## 5 Conclusions

1. In bilateral and unilateral listening, compared to the FS4 strategy, the MOC1 and MOC_FE_ improved the intelligibility of speech in fluctuating noise but not in stationary noise. This is consistent with the findings of our own previous studies. The greater benefit in fluctuating than in stationary noise is likely due to the use of fast time constants of binaural MOC control.
2. The speech-in-noise intelligibility benefits provided by the MOC_FE_ strategy (relative to the FS4) are similar as those provided by the MOC1 strategy for the S_0_N_0_, S_0_N_90_, S_−15_N_15_ spatial configurations. For the S_−60_N_60_ configuration, however, the MOC_FE_ strategy provided smaller benefits than the MOC1 strategy possibly because contralateral inhibition was stronger with MOC_FE_ strategy and reduced audibility too much. The issue might be solved by optimizing the parameters of the MOC_FE_ strategy.
3. In bilateral and unilateral listening, all tested strategies produced spatial release from masking.
4. Independently of the stimuli used, the MOC1 and MOC_FE_ strategies tended to improve sound source localization slightly relative to the FS4 strategy.
5. The present findings suggest that it may be possible to implement the binaural MOC strategy in the frequency domain and at the front end rather than the back end of processing.

## Data Availability

The raw data supporting the conclusions of this article will be made available by the authors, without undue reservation.
